# Identification of double-yolked duck egg using computer vision

**DOI:** 10.1371/journal.pone.0190054

**Published:** 2017-12-21

**Authors:** Long Ma, Ke Sun, Kang Tu, Leiqing Pan, Wei Zhang

**Affiliations:** 1 College of Food Science and Technology, Nanjing Agricultural University, Nanjing, Jiangsu, People's Republic of China; 2 College of Food and Biological Engineering, Bengbu University, Bengbu, Anhui, People's Republic of China; 3 College of Food Science, Nanjing Xiaozhuang University, Nanjing, Jiangsu, People's Republic of China; Plymouth University, UNITED KINGDOM

## Abstract

The double-yolked (DY) egg is quite popular in some Asian countries because it is considered as a sign of good luck, however, the double yolk is one of the reasons why these eggs fail to hatch. The usage of automatic methods for identifying DY eggs can increase the efficiency in the poultry industry by decreasing egg loss during incubation or improving sale proceeds. In this study, two methods for DY duck egg identification were developed by using computer vision technology. Transmittance images of DY and single-yolked (SY) duck eggs were acquired by a CCD camera to identify them according to their shape features. The Fisher’s linear discriminant (FLD) model equipped with a set of normalized Fourier descriptors (NFDs) extracted from the acquired images and the convolutional neural network (CNN) model using primary preprocessed images were built to recognize duck egg yolk types. The classification accuracies of the FLD model for SY and DY eggs were 100% and 93.2% respectively, while the classification accuracies of the CNN model for SY and DY eggs were 98% and 98.8% respectively. The CNN-based algorithm took about 0.12 s to recognize one sample image, which was slightly faster than the FLD-based (about 0.20 s). Finally, this work compared two classification methods and provided the better method for DY egg identification.

## Introduction

The occurrence of double-yolked (DY) avian eggs is a common phenomenon in commercial species of poultry, waterfowl, and game birds [1–6]. DY eggs are formed when two yolks ovulated within three hours of each other become enclosed in one egg [7, 8], and estimated to occur in 4~12.5% of broiler breeder pullet eggs in the first 3 months of laying [9, 10], 1.1~3.5% of laying hen eggs [11], and 2~10% of Gaoyou duck (an indigenous Chinese breed) eggs [12].

DY eggs are considered as a loss to overall commercial hatcheries because of their relatively lower yolk fertility rate due to their smaller yolk size and markedly lower hatchability rate due to lack of space to move compared to single-yolked (SY) eggs [4, 6, 13–15]. These unhatched eggs are waste of space and energy in the incubator, and have the potential to contaminate other hatching eggs by infecting them with bacteria or molds, thus DY eggs are mostly removed before incubation in commercial hatcheries [16, 17].

Although DY eggs are not suitable for using as hatching eggs, they have great edible and commercial values. The absolute quantities of the nutrient components are greater in DY eggs as they are physically larger than SY eggs but the nutrient’s relative proportions also different from those in SY eggs [6, 18]. According to Chinese and Japanese folklore, DY eggs are believed to be a sign of good luck [12]. Therefore, consumers in China and Japan prize DY eggs above SY eggs. DY eggs are roughly 6~8 times more expensive than SY eggs in the Chinese market [19, 20].

Commercial hatcheries exclude DY eggs from hatching in order to decrease egg loss during incubation, while egg producers pick out them for sale to improve economic returns. Therefore, an accurate, nondestructive, and automatic method is needed to improve the DY egg identification in the poultry industry. Computer vision, which uses computers to emulate human vision, is a detection technology with the advantages of high automaticity, high detection efficiency, low cost, and easy maintenance [21–23]. Computer vision technology has been successfully applied to the rapid identification of animal and plant products, such as cichlid fishes [24], grains [25], and field peas [26].

The traditional method for DY egg identification is “candling” and involves illuminating a bright light through each egg. By sending light through the DY egg, two rounded gray shadows (the yolks) become visible. Since candling is a visual process, it is possible to use a computer vision system to accomplish the identification of DY eggs. Wang *et al*. [27] developed the first computer vision system to identify DY chicken eggs. They used the geometrical characteristics (i.e., egg area, yolk area to perimeter ratio, yolk area to egg area ratio, yolk perimeter to egg perimeter ratio) from digital images to detect DY eggs from SY eggs at a correct recognition rate of over 95%. The judgment criterion they used was based on the statistical conclusion that DY eggs were larger than SY eggs. In reality, some DY duck eggs are similar in size to SY eggs and such eggs are always produced at the onset of laying [15]. These geometric characteristics would be not sufficient to identify DY eggs and carry a considerable risk of failure. Further, this method was suitable only for identifying certain preassigned egg species.

In the countries of China and South East Asia, duck egg consumption, either as fresh or preserved, accounts for about 30% of the total egg consumption [28, 29]. Giving rise to the need to identify DY duck eggs. This study investigated the potential of using computer vision to classifying duck eggs into SY and DY categories. For this purpose, the research was conducted through (1) to establish an image preprocessing algorithm that could be used to isolate the regions of interest (ROIs) from sample images; (2) to extract shape descriptors of yolk, and apply them to build a Fisher’s linear discriminant (FLD) model to separate SY from DY eggs; (3) to build a convolutional neural network (CNN) model fed with primary preprocessed images; and (4) to compare the classification accuracy rates and wall clock time of the two models and thereby determine the better method for DY duck egg identification. The final goal of this work is to develop the system for an on-line grading machine (utilized on the conveyor belts) in the industry, for the future.

## Materials and methods

### Duck egg materials

Eggs from a flock of Shaoxing ducks (*Anas platyrhynchosdomesticus*) which were within two days of being laid were obtained directly from a local poultry farm in the Pukou district of Nanjing City, Jiangsu Province, China, over a 5-month period (between March and July 2016). A total of 500 DY and 500 SY eggs were identified by well-trained local workers at the farm via hand candling. Small and large DY duck eggs (termed by Salamon and Kent [15]) were collected in this study. Once eggs arrived at the laboratory, they were washed to remove the dirt and cuticle from their surface and then quickly dried and individually numbered prior to further testing.

### Image acquisition

**Fig 1:**
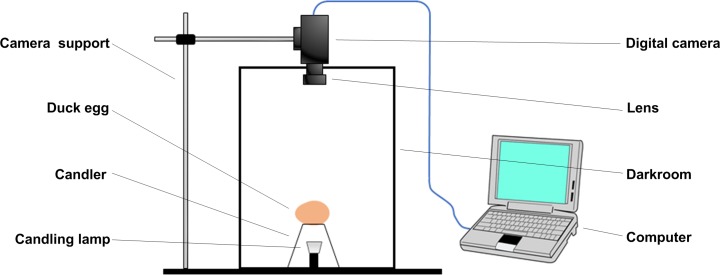
Self-made computer vision system.

**Fig 2:**
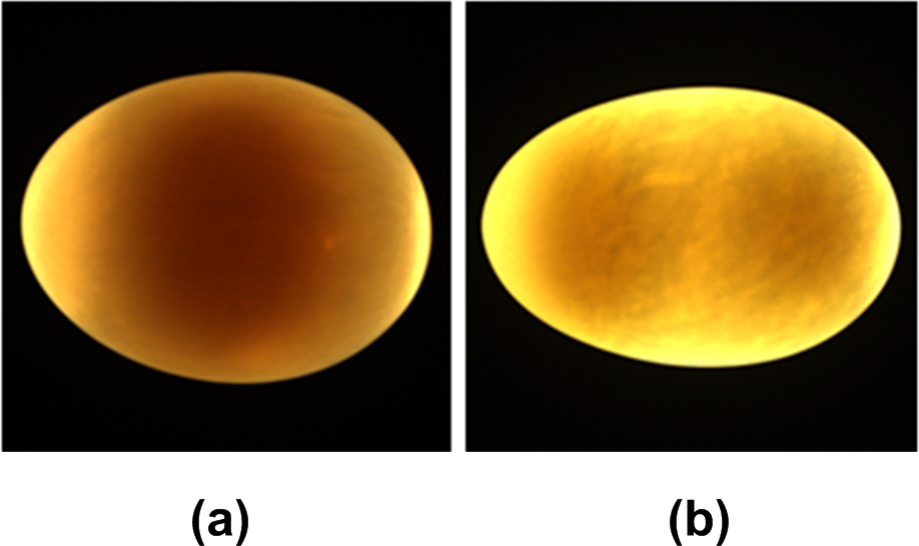
Typical color images of duck egg samples. (a) From SY egg. (b) From DY egg.

To acquire clear transmittance images of duck eggs, a self-made computer vision system was developed, as shown in Fig 1. This system adopted an Imaging Source color CCD camera (model: DFK23U274) equipped with a manually controlled Pentax TV lens (focal length 16 mm; maximum shutter aperture 1.4). The light source in the egg candler was a LED lamp with a nominal voltage of 12 V, a rated power of 5 W, and a color temperature of 3500 K. To reduce any disturbance to the light from the external environment, the image capture was done in a closed darkroom, a wooden cubic chamber 40×40×50 cm in size and painted black on the inside. The top of the egg candler and the camera lens were set 35 cm apart. During the image capture, the duck egg was set horizontally on the egg candler and an image was taken with an aperture value of 4.0 and exposure of 1/10 s. The resulting images (Fig 2) were saved in BMP format with a resolution of 1600×1200 pixels.

### Image preprocessing

**Fig 3:**
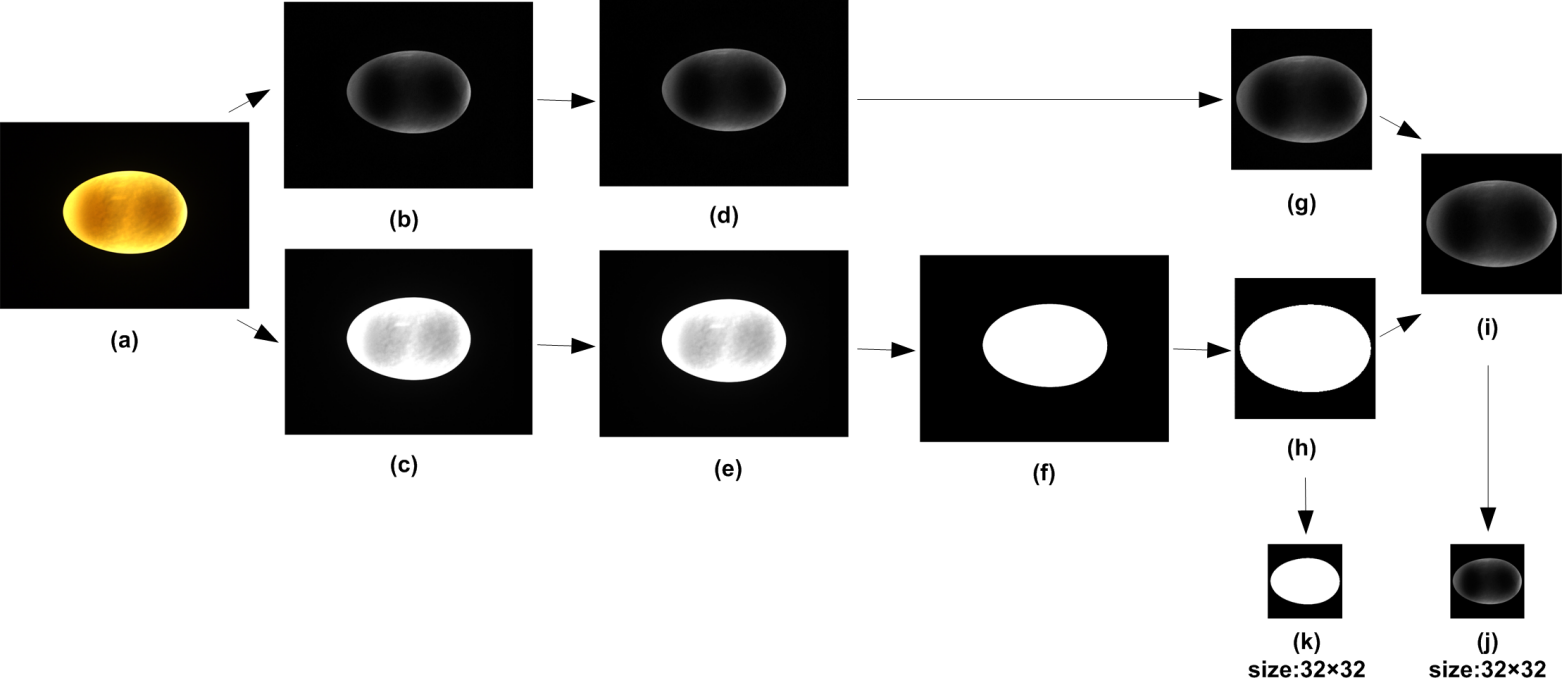
Flow chart of the image preprocessing algorithm. (a) Original color image. (b) B channel separating. (c) R channel separating. (d) Image de-noising for B channel. (e) Image de-noising for R channel. (f) Threshold segmentation for R channel. (g) ROI cropping. (h) Mask cropping. (i) Background removal for ROI. (j) ROI resizing. (k) Mask Resizing.

The image preprocessing algorithm was developed in order to isolate ROIs from their respective backgrounds with Matlab software, as shown in Fig 3.

#### Color channel separating

RGB color space is oriented toward hardware (such as for color monitors and a broad class of color video cameras) [30]. The images captured by the CCD camera used in this study are the RGB images. The R, G, and B channel images were separated from each original image (Fig 3A). It was easier to see the differences in gray values between the yolk area and the surroundings in the B channel image (Fig 3B). Therefore, yolk region could be isolated from this channel. There was a clear contrast between the background and the egg itself in the R channel image (Fig 3C). Thus, this channel provided better outline information of egg region.

#### Image de-noising

A 3×3 median filter was used to de-noise the R and B channel images separately to remove noise and reduce edge signal distortions in the images (Fig 3D and 3E).

#### ROI cropping

The objective of this sub-process was to locate the egg in the original image and obtain ROI’s cropping coordinates. The segmentation was done after thresholding the de-noised R channel image with the Otsu’s algorithm [31]. This generated a binary image (Fig 3F) where the pixels of the duck egg were white (gray level = 1) and the pixels of the background were black (gray level = 0). The pixel coordinates of four vertices of the minimum enclosing square of egg outline were calculated with the *regionprops* function. To prevent over-cropping or loss of edge information, the square region was extended in four directions by 30 pixels to form the ROI’s coordinates. Finally, the *imcrop* function was applied to crop a square matrix from the B channel image after de-noising and the ROI was created (Fig 3G).

#### Background removal

The binary duck egg image (Fig 3F) was cropped with the ROI’s cropping coordinates described above to obtain a mask image (Fig 3H). The resulting ROI was then multiplied by the mask image to obtain a new ROI (Fig 3I) wherein the background was pure black (gray level = 0) and the gray level of egg region remained unchanged [32].

#### Image size normalization

The dimensions of the new ROI were resized into a 32×32 matrix (Fig 3J) using bicubic interpolation owing to variations in the size of each sample [33]. The mask image was subject to the same changes (Fig 3K).

### FLD-based algorithm development

FLD was chosen as the classification method for classifying DY and SY eggs as a result of its relative simplicity and lower computational cost compared to other classification methods [26]. FLD as a conventional machine learning technology requires firstly manually designing the feature vectors and extracting them.

#### Morphological characteristics extraction

**Fig 4:**
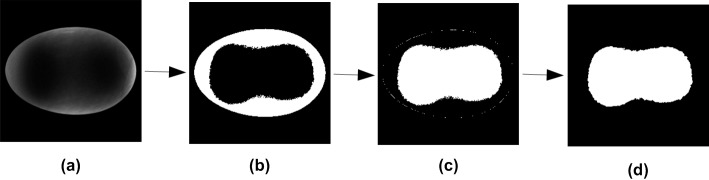
Flow chart of morphological characteristics extraction algorithm. (a) ROI image. (b) Threshold segmentation. (c) Subtraction operation. (d) Eggshell boundary removal.

The shape features of an object can be categorized based on its morphological characteristics in a digital image. The image processing steps were developed to extract the morphological characteristics of egg yolk as shown in Fig 4. Firstly, a binary image (Fig 4B) was generated after thresholding the resized ROI with the Otsu’s algorithm. In this binary image, the albumen region was white while the yolk region and the background were black. Secondly, a subtraction operation was performed between the pixel values of the resized mark image (Fig 3K) and Fig 4B (i.e., Fig 3K minus Fig 4A). In the resulting binary image (Fig 4C), the yolk region and eggshell boundary appeared in white. Finally, a 3×3 median filter was used to treat the image obtained above in order to remove the eggshell boundary. After that, the final binary image (Fig 4D) contained only the yolk region, where white pixels (active pixels, gray level = 1) represented morphological characteristics of egg yolk and black pixels represented the background (gray level = 0).

#### Shape feature extraction

Fourier Descriptors (FDs) conceptualized by Zahn and Roskies [34] were used to describe the yolk shape in this study. FD, one of shape descriptors, has been popular for sharp recognition, matching and registration purposes[35–38]. Following the method described by Gonzalez [30], a set of complex Fourier coefficients (i.e., Fourier Descriptors) were generated by the discrete Fourier transform (DFT) of the yolk boundary.

FDs represent the shape of an object in a frequency domain. In theory, the harmonic orders of FDs range from 0 to *N*-1 (where *N* is the number of boundary points). However, it is one favorable property common to FDs that an approximate boundary can be reconstructed using only a few low-frequency coefficients. For classification purposes, only the first few low-frequency FDs were generally enough to distinguish the difference between yolks shapes.

To eliminate the impacts of the translation, rotation, scale of the shapes, and starting point of the outline trace, the Fourier coefficients were normalized using the method of Yadav *et al*. [39]. With this procedure, the binary image of yolk boundary was automatically transformed into the translation, scale and rotation invariant FDs, i.e., normalized Fourier descriptors (NFDs).

#### FLD model development

The duck eggs were first photographed with a CCD camera. Immediately, the NFDs were extracted from the sample images and used as feature vectors. Then, each egg was opened manually to confirm the actual yolk type. The NFDs were used as input variables, and the manual recognition results were used as output classes. The FLD model was built and analyzed in IBM SPSS v20.0 software. After a set of Fisher discriminant functions are calculated, they can be applied to identify an unknown sample with the codes written in Matlab language.

### CNN-based algorithm development

CNNs are feedforward, backpropagate neural networks with a special architecture inspired from the visual system [40]. CNN, as one of the most popular deep learning models at present, has led major advances in the computer vision research community. Compared to conventional machine learning technologies, such as FLD, support vector machine (SVM) and back-propagation neural network (BPNN), CNN allows the net to be fed with raw or only minimally preprocessed images so as to automatically learn the image features needed for recognition and achieves a higher accuracy in practical applications [41].

Prior to treating the original images via CNN, the sample images were subjected to several initial pre-processing operations eliminating redundant information in order to shorten the wall clock time of the algorithm. The B channel images were first extracted from the color sample images since the shape features were the criterion for identifying DY eggs, rather than the color features. The ROI’s dimensions were then reduced to a 32×32 matrix after 3×3 median filtering, cropping, and background removal with the methods described above. These small preprocessed ROIs after being normalized (i.e., divided by 255) were used as input data, and the types of samples (SY eggs labeled [0, 1] and DY eggs labeled [1, 0]) were used as labels. The CNN model was built and validated in Matlab R2012b software with DeepLearnToolbox-master toolbox (developed by Rasmus Berg Palm, downloaded from https://github.com/rasmusbergpalm/DeepLearnToolbox).

## Results

### Feature vector selection for FLD model

**Fig 5:**
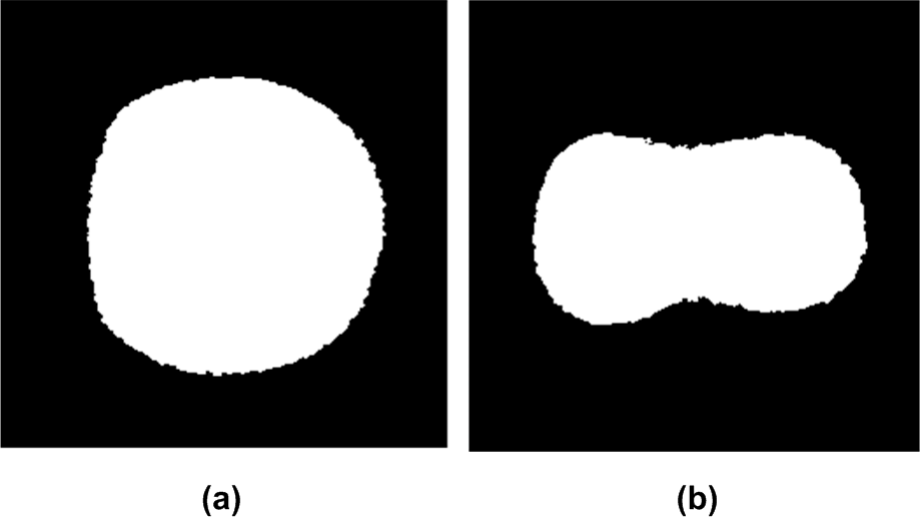
Two typical binary yolk images. (a) From SY egg. (b) From DY egg.

All the 1000 original images of duck eggs automatically were converted to binary yolk images with smooth boundaries after being subjected to the image processing sequence. Typical binary yolk images of SY and DY eggs are shown in Fig 5. The SY egg yolk image was roughly circular in shape, while the DY egg’s was shaped like an “8”.

**Fig 6:**
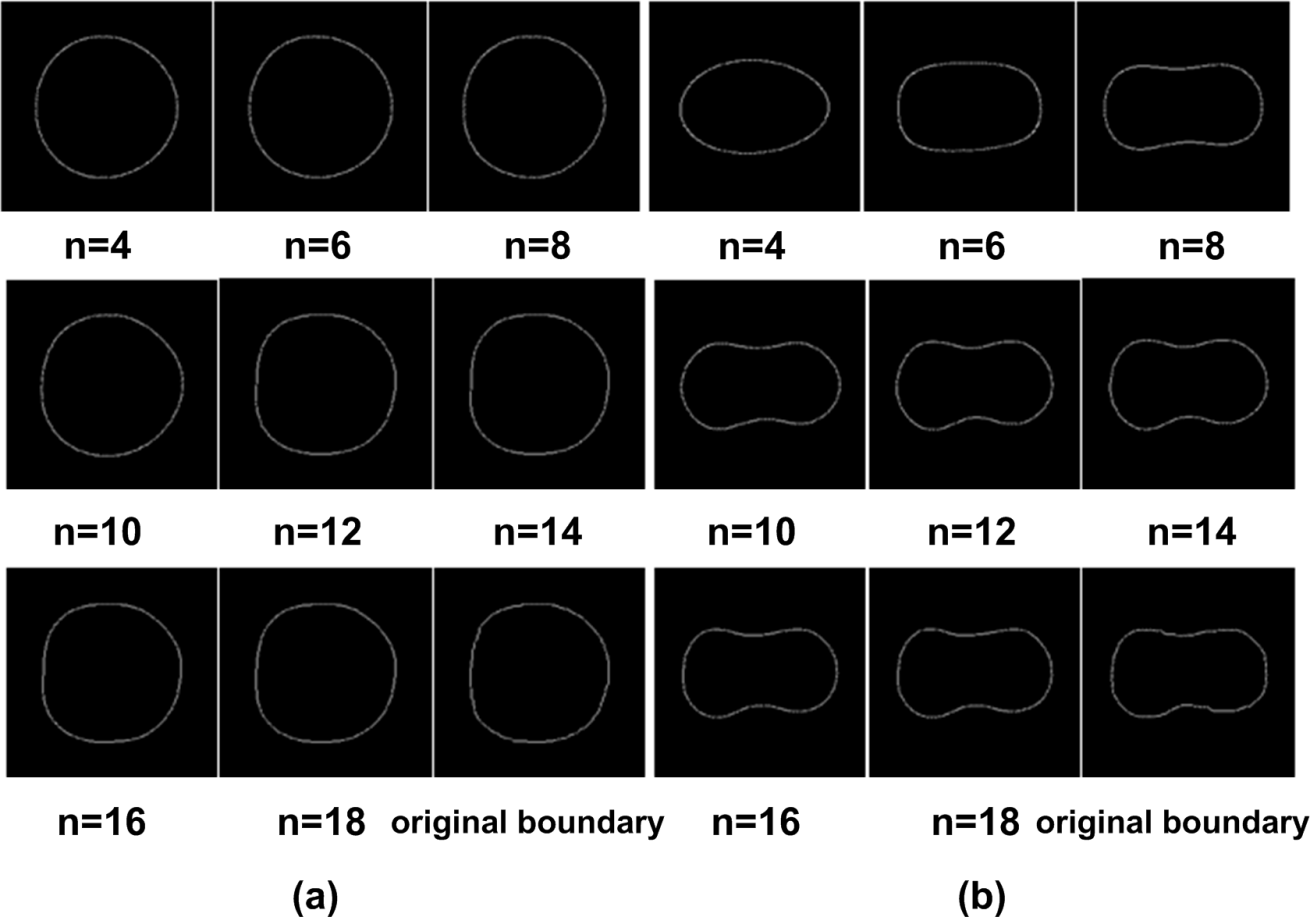
DY and SY egg yolk boundaries reconstructed from the first 4, 6, 8, 10, 12, 14, 16, and 18 FDs and the original boundaries. (a) From SY egg. (b) From DY egg.

The DY and SY duck egg yolk boundaries reconstructed from the first 4, 6, 8, 10, 12, 14, 16, and 18 FDs are shown in Fig 6. The reconstructed boundaries grew closer to the original boundaries as the number of FDs increased. Moreover, the first 16 FDs were considered appropriate for approximating the DY and SY duck egg yolk boundaries.

**Fig 7:**
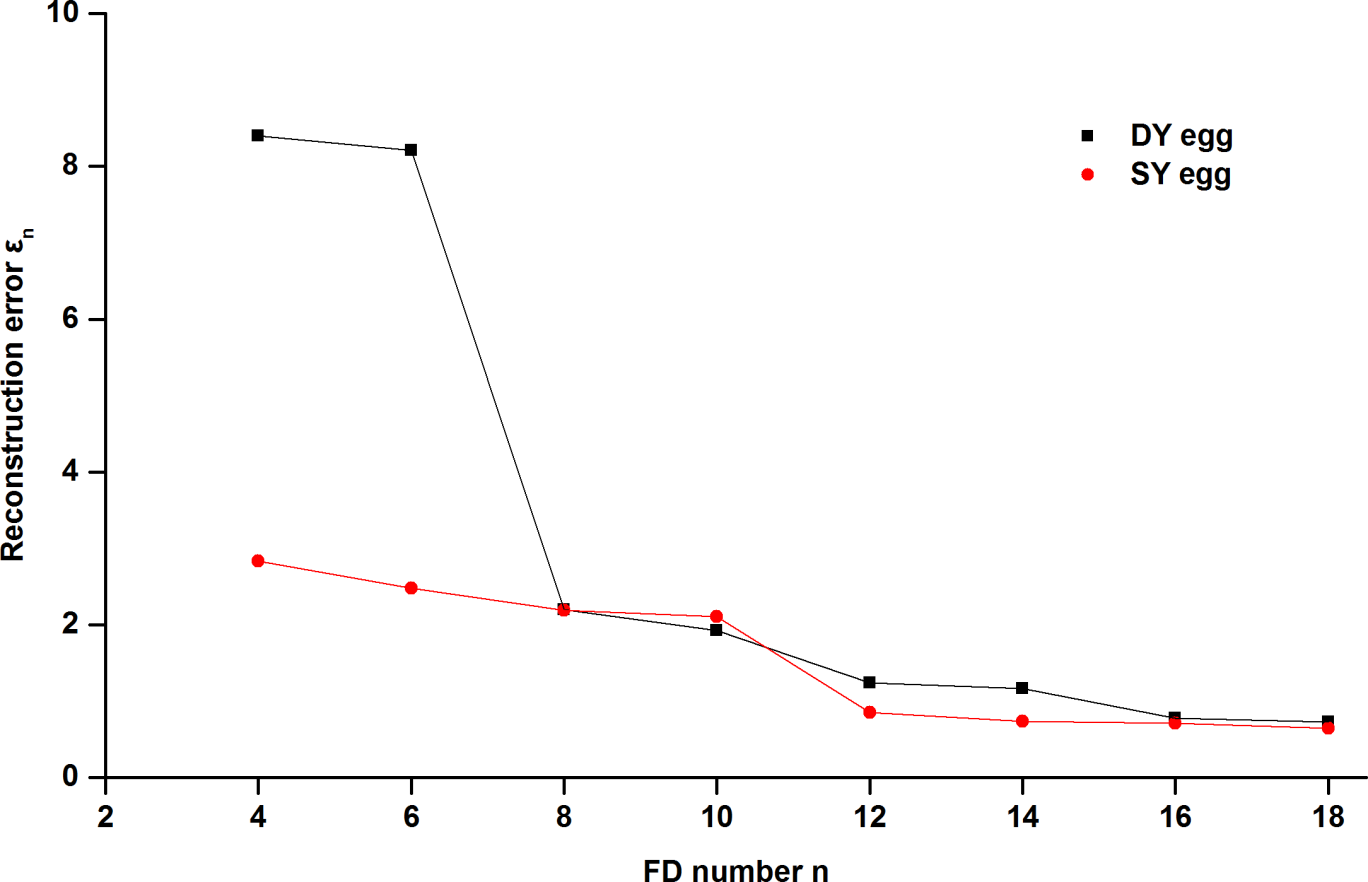
Reconstruction error versus the number of FDs.

To come to a more objective conclusion regarding the relationship between the actual error of the reconstructed boundary compared to the original and the FD quantity, 20 DY and 20 SY duck egg images were randomly selected as test objects to calculate their reconstruction errors *ε*_*n*_ from Eq 1 [42] using different quantities of FDs. Fig 7 shows the relationship between the reconstruction error *ε*_*n*_ and the FD quantity. The reconstruction errors of the two type eggs both decreased rapidly as the number of FDs increased and became eventually negligible after the 16th FD.

$$\varepsilon_{n}=\frac{1}{N}\sum_{k=0}^{N-1}\min_{k\in C}\left(\sqrt{{\left(x_{k}-x_{nk}\right)}^{2}+{\left(y_{k}-y_{nk}\right)}^{2}}\right)$$ (1)

where *ε*_*n*_ is the error between the reconstruction and original boundary based on the first *n* harmonics, *N* is the number of the points on the original boundary, (*x*_*k*_, *y*_*k*_) are the original boundary coordinates for point *k* (*k* = 0, 1, 2, …, *N*-1), and (*x*_*nk*_, *y*_*nk*_) are the reconstruction coordinates respectively corresponding to (*x*_*k*_, *y*_*k*_).

The reconstructed boundary images and error curves altogether indicated that the coefficients of the first 16 low-frequency harmonics could accurately approximate the original yolk boundary. After the first 16 FDs being normalized, the first two of the resulting NFDs *d*(*u*) (for *u* = 0, 1, 2, …, *N*-1, where *u* is the harmonic order and *N* is the number of boundary points) were constants (i.e., *d*(0) = 0 and *d*(1) = 1), therefore they were discarded. Ultimately, *d*(2)-*d*(15) were chosen from the original data to constitute the 14-dimensional feature vector [*d*(2), *d*(3), …, *d*(15)] as the input variables to build the classification models.

### FLD model for identifying duck egg yolk types

The FLD model was built based on all 500 DY and 500 SY duck egg images. Consequently, a set of Fisher classification functions for classifying the types of duck eggs were obtained. This model was validated using the leave-one-out cross-validation method. The performance of the FLD model is outlined in Table 1. No sample from SY eggs was misclassified and the misclassified samples were from DY eggs. The classification accuracies for SY and DY eggs were 100% and 93.2% respectively.

**Table 1:** Classification results for duck egg yolk type using FLD.

	Group	Predicted Group Membership		Total
		SY eggs	DY eggs	
**Case selected**				
***Original*^a^**				
**Count**	**SY eggs**	500	0	500
	**DY eggs**	34	466	500
**%**	**SY eggs**	100	0	100
	**DY eggs**	6.8	93.2	100
***Cross-validated*^b^**				
**Count**	**SY eggs**	500	0	500
	**DY eggs**	34	466	500
**%**	**SY eggs**	100	0	100
	**DY eggs**	6.8	93.2	100

^a^ Each case was classified by the functions derived from all cases.

^b^ Cross validation was done only for those cases in the analysis. In cross validation, each case was classified by the functions derived from all cases other than that case.

### CNN Model for identifying duck egg yolk types

**Fig 8:**
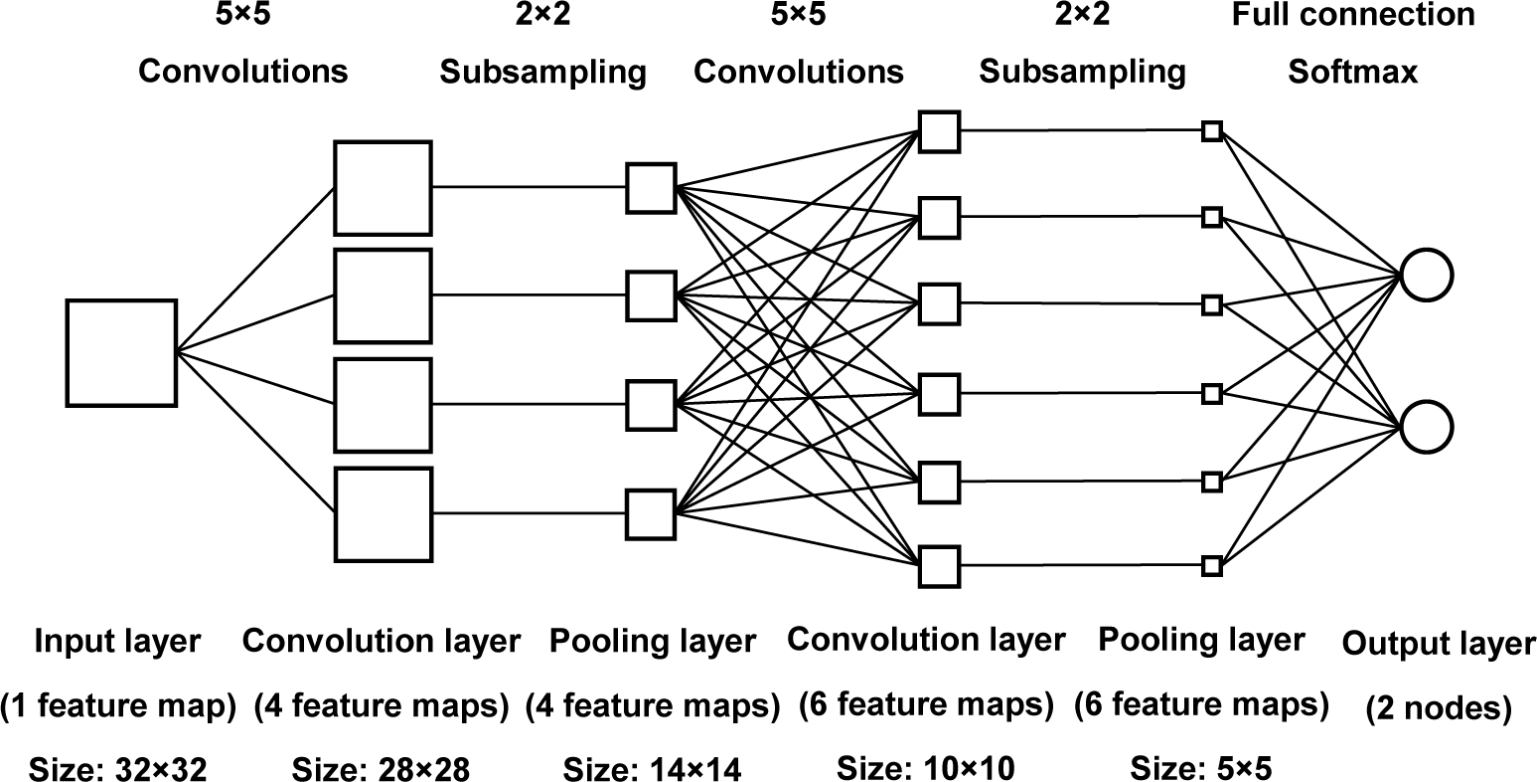
The structure of CNN model built for recognition of each egg yolk image type.

The CNN model was used to recognize all the sample images. The network was created following the traditional LeNet architecture (developed by LeCun [40, 43], who invented the convolution networks). The structure of this CNN model is shown in Fig 8. This model consisted of one input layer, two pairs of alternating layers of convolution layers and average pooling layers, and one fully connected output layer. The input layer had a 32×32 neuron array to receive the small preprocessed ROIs from the original sample images. The first convolution and average pooling layers each had four feature maps and the second groups each had six. The size of the convolution kernel was set to 5×5, and the size of the region was set to 2×2 to pool the convolved features over. The second convolution layer was all connected to the first average pooling layer. The output layer consisted of two output neurons corresponding to the two class labels. The feature maps of the last average pooling layer concatenated into a column vector which fed into the output layer. The sigmoid function was used as activation function at the convolution and pooling layers, and softmax function was used as activation function for the recognition problem at the final output layer.

All the sample images were randomly divided into two groups at a ratio of 95:5, each of which contained the same number of DY and SY eggs. One group was used as the training set and the other as the validation set. The CNN model was trained on the training set, and then the trained CNN was applied on the validation set to measure the accuracy. The experiment above was repeated 10 times, and the accuracies were recorded and averaged. The CNN models were trained using Stochastic Gradient Descent (SGD) with a fixed learning rate of 0.2 and a batch size of 50 for 100 training epochs. The average classification accuracies of the CNN model for SY and DY eggs were 98% and 98.8% respectively. A few of samples from SY eggs were misclassified besides a few of DY egg samples. For detailed information, please refer to S2 Table.

### Wall clock time with different classification methods

**Fig 9:**
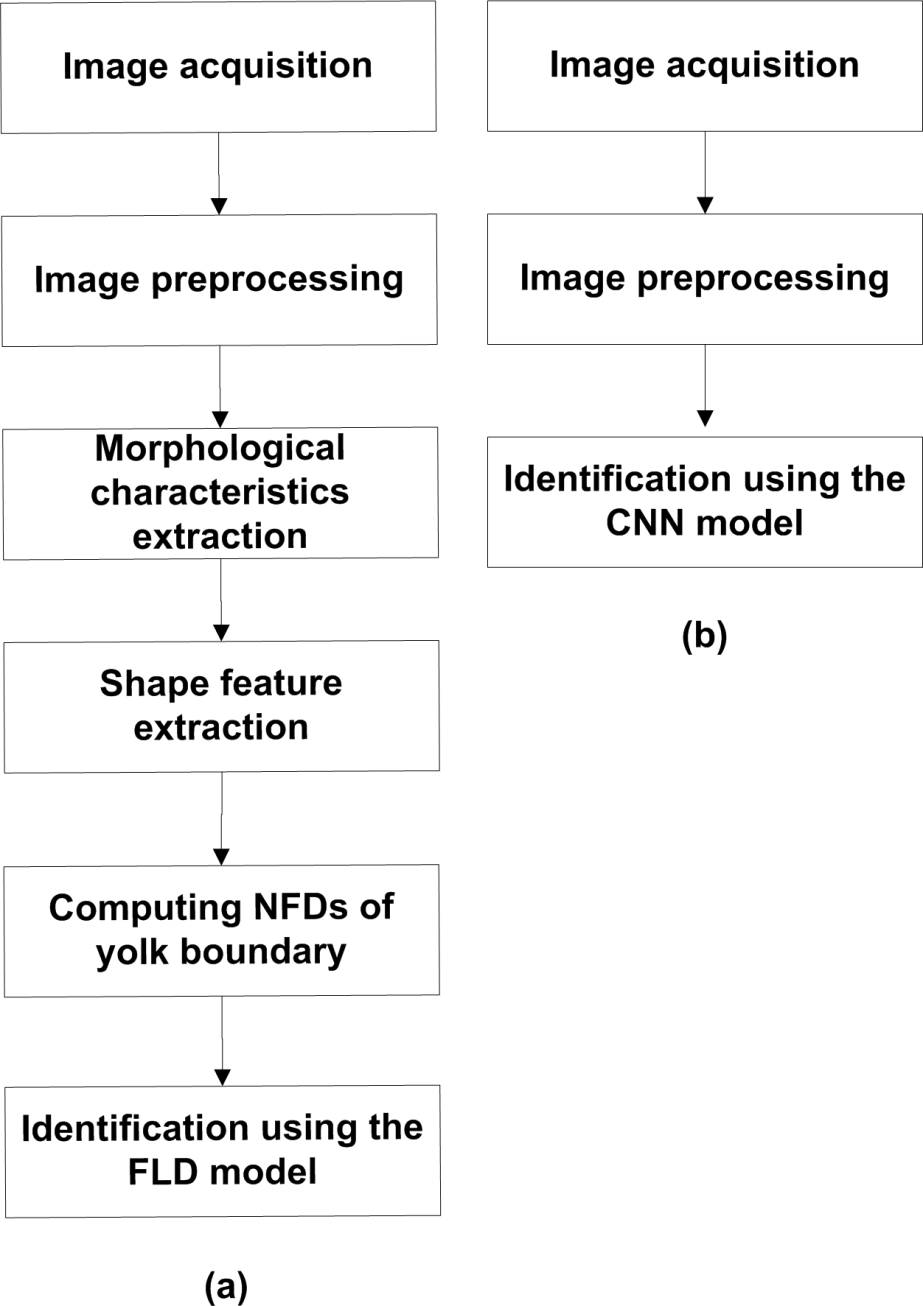
Flow chart of DY egg identification algorithms. (a) The FLD-based algorithm. (b) The CNN-based algorithm.

25 DY and 25 SY egg images were randomly chosen from the sample images and used as test objects to measure the execution speed (wall clock time) of two whole algorithms based on FLD and CNN models, shown in Fig 9. The codes for the two algorithms were both written using Matlab language. The wall clock time was calculated by employing a stopwatch timer function (based on the *tic* and *toc* functions) [44]. All tests were performed on a computer equipped with an Intel Core i7-3537U @ 2.00 GHz processor, 8 GB RAM, Windows 7 system with Matlab R2012b software.

The code for each algorithm was run 10 times, and the wall clock time from the image preprocessing to the sample identification was recorded and averaged. The average wall clock time of the FLD- and CNN-based algorithms to recognize one sample image was 0.20±0.0075 s and 0.12±0.0080 s, respectively. For detailed value, please refer to S3 Table. The FLD-based algorithm spent much more time recognizing one sample image than the CNN-based. This was mainly because the former needed to extract complex characteristic parameters from sample image while the latter did not. Comparing the accuracy rates and wall clock time of the two methods, it was concluded that the CNN-based method was the better method for identifying DY duck eggs.

## Discussion

In commercial hatcheries and egg producers, candling is a reliable and unique technique for DY egg identification. The inspection of eggs for double yolks is a major bottleneck because it is largely done by human workers. Candling suffers from judgment errors due mainly to human subjectivity, visual stress, and tiredness, especially when linked with high-speed grading machines. In the last two decades, many researchers have attempted to design and develop computer vision systems to replace human operators for egg quality assessment. In this study, computer vision technology was applied to the identification of DY duck eggs. Compared to candling, computer vision technology can control costs, reduce the workload on workers, and increase the efficiency, accuracy, and stability of the yolk identification process.

In this paper, the FLD-based method for DY duck egg identification was developed using computer vision and conventional machine learning technology. Compared to Wang’s method [27], this method was simple and accurate. The morphological characteristics of egg yolks were first extracted from sample images, and then the shape features (i.e., FDs) were calculated to establish a FLD model. The model worked based on the fact that there is a substantial visual difference in the yolk shapes in SY and DY egg images.

**Fig 10:**
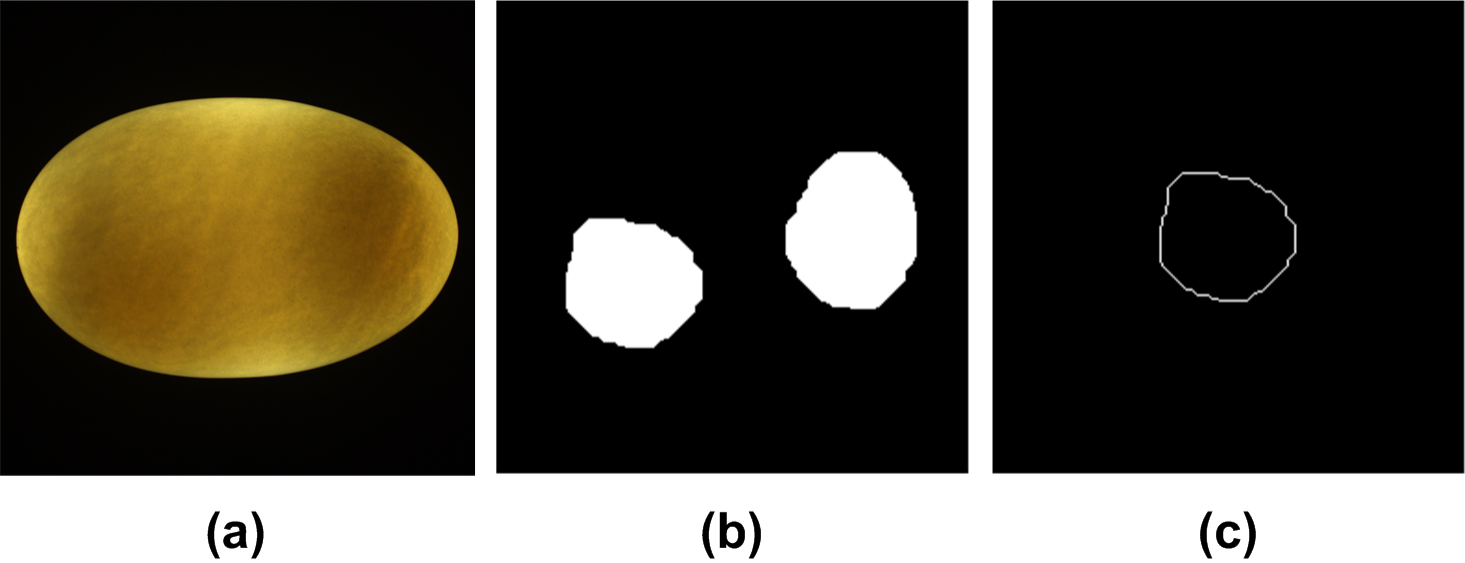
Typical yolk image with two separate yolk regions. (a) Original color image. (b) Yolk binary image. (c) Yolk boundary image using the method described by Gonzalez [30].

However, the FLD-based method was ineffective for a few sample images where the two yolk regions were separated (shown in Fig 10). There were two separate closed boundary curves in these sample images. FDs could be calculated from single closed curve [34], so only the first connected component was extracted to obtain FDs. As described above, the yolk region was segmented after thresholding with the Otsu’s method (one of global thresholding techniques). The size of the yolk region depended on the threshold level. The computation of the threshold level using Otsu’s method is mainly impacted by the gray level distribution of the image [30]. The inhomogeneity of egg sample itself caused by eggshell light transmittance made the threshold of the captured sample image away from the optimal threshold for yolk segmentation. If the threshold calculated here was too large, the yolk regions were relatively small and the two yolk regions were separated.

In this study, the CNN-based method demonstrated better performance for identifying DY eggs compared to the FLD-based. CNN, as a deep learning technology, can self-learn deep visual features, and has advantages in both classification ability and speed. CNN has already been successfully applied in recognizing animals and plants such as dairy cows [45] and Chinese herbal medicines [46]. Grayscale images from the B channel were used as inputs to the CNN network to identify DY eggs. The CNN-based method developed here showed slightly shorter wall clock time than the FLD-based and was able to correctly identify most of the sample images where the two yolk regions underwent segregation. In short, the CNN-based method was better for automatic DY egg detection. Samples we used in our experiments were within two days of being laid, but if they are burst yolks or scattered single yolks caused by unavoidable vibration during the transportation, they will also be occasionally misclassified to double yolks because their image characteristics are similar.

## Conclusions

This study was conducted in an effort to develop a set of techniques for separating DY from SY duck eggs using computer vision. Both the FLD and CNN model were investigated for duck egg type identification. Training and validation of the FLD model were performed using NFDs extracted from transmittance images of duck egg samples, while those of the CNN model were performed using grayscale images. The classification accuracies of the FLD model for SY and DY eggs were 100% and 93.2% respectively, while the classification accuracies of the CNN model for SY and DY eggs were 98% and 98.8% respectively. The CNN-based algorithm took about 0.12 s to recognize one sample image, which was slightly faster than the FLD-based (about 0.20 s). Both methods can be adapted to the real-time detecting process. The CNN-based method has a better prospect of application, although its algorithm has higher requirement to computer hardware than the FLD-based. The results of this study effectually lay a foundation for the further development of industrial, automatic DY egg sorting equipment based on computer vision.

## Supporting information

S1 Table The corresponding value of data for Fig 7. (PDF)

S2 Table The confusion matrices of the validations repeated 10 times. (PDF)

S3 Table The wall clock time of the FLD- and CNN-based algorithms. (PDF)
