# Supplementary material for: Identification of double-yolked duck egg using computer vision
Source: PLoS One. 2017 Dec 21;12(12):e0190054. doi: 10.1371/journal.pone.0190054 (PMC5739493; doi:10.1371/journal.pone.0190054)
Supplement: S1 Table — (PDF) [file pone.0190054.s001.pdf]

S1 Table. The corresponding value of data for Fig 7.

ε_n	**4 FDs**	**6 FDs**	**8 FDs**	**10 FDs**	**12 FDs**	**14 FDs**	**16 FDs**	**18 FDs**
**SY eggs**	2.8365	2.4846	2.1918	2.1129	0.8522	0.7438	0.7149	0.6531
**DY eggs**	8.3952	8.2038	2.2031	1.9308	1.2448	1.1695	0.7804	0.7360
