# Supplementary material for: Identification of double-yolked duck egg using computer vision
Source: PLoS One. 2017 Dec 21;12(12):e0190054. doi: 10.1371/journal.pone.0190054 (PMC5739493; doi:10.1371/journal.pone.0190054)
Supplement: S2 Table — (PDF) [file pone.0190054.s002.pdf]

S2 Table. The confusion matrices of the validations repeated 10 times.

The first time		Actual Group Membership	
		SY eggs	DY eggs
Predicted Group	SY eggs	25	0
Membership	DY eggs	0	25

The second time		Actual Group Membership	
		SY eggs	DY eggs
Predicted Group	SY eggs	24	0
Membership	DY eggs	1	25

The third time		Actual Group Membership	
		SY eggs	DY eggs
Predicted Group	SY eggs	25	0
Membership	DY eggs	0	25

The fourth time		Actual Group Membership	
		SY eggs	DY eggs
Predicted Group	SY eggs	24	1
Membership	DY eggs	1	24

The fifth time		Actual Group Membership	
		SY eggs	DY eggs
Predicted Group	SY eggs	25	0
Membership	DY eggs	0	25

The sixth time		Actual Group Membership	
		SY eggs	DY eggs
Predicted Group	SY eggs	25	1
Membership	DY eggs	0	24

The seventh time		Actual Group Membership	
		SY eggs	DY eggs
Predicted Group	SY eggs	24	0
Membership	DY eggs	1	25

The eighth time		Actual Group Membership	
		SY eggs	DY eggs
Predicted Group	SY eggs	24	0
Membership	DY eggs	1	25

The ninth time		Actual Group Membership	
		SY eggs	DY eggs
Predicted Group	SY eggs	24	1
Membership	DY eggs	1	24

The tenth time		Actual Group Membership	
		SY eggs	DY eggs
Predicted Group	SY eggs	25	0
Membership	DY eggs	0	25
