# Supplementary material for: Identification of double-yolked duck egg using computer vision
Source: PLoS One. 2017 Dec 21;12(12):e0190054. doi: 10.1371/journal.pone.0190054 (PMC5739493; doi:10.1371/journal.pone.0190054)
Supplement: S3 Table — (PDF) [file pone.0190054.s003.pdf]

S3 Table. The wall clock time of the FLD- and CNN-based algorithms.

The CNN-based algorithm											
		The wall clock time (s)									
		1	2	3	4	5	6	7	8	9	10
DY eggs	1	0.117917017	0.117726124	0.119424862	0.116270416	0.120240568	0.134061179	0.121564909	0.138292012	0.121052168	0.122846557
	2	0.129122197	0.123015281	0.122529635	0.122265669	0.120410934	0.119336189	0.113462039	0.14399949	0.133557059	0.122266901
	3	0.126089676	0.121492658	0.120197052	0.139721036	0.120924086	0.125099498	0.111811744	0.118429758	0.12119503	0.119522155
	4	0.122979155	0.113393893	0.122359268	0.117706009	0.132417341	0.117215436	0.122283732	0.124236993	0.111921763	0.124246845
	5	0.145286474	0.112520714	0.139415609	0.139331862	0.125726364	0.116575023	0.145402652	0.120091138	0.118248718	0.121350617
	6	0.15074846	0.128057715	0.114708382	0.122292353	0.124649156	0.118118583	0.126387304	0.124126973	0.127954263	0.122985724
	7	0.119922414	0.122429878	0.119479051	0.113589301	0.121519752	0.119189222	0.12603836	0.121204061	0.119699911	0.115078672
	8	0.140995705	0.120490165	0.118694955	0.124554736	0.142346731	0.117702314	0.123850282	0.124475095	0.122505824	0.121631003
	9	0.134628519	0.113821656	0.12202182	0.119972497	0.142382446	0.118568925	0.120784508	0.120082927	0.137839617	0.115593466
	10	0.115853326	0.120751256	0.120205673	0.115386974	0.136761588	0.120010676	0.121984873	0.121947926	0.122714369	0.118961794
	11	0.125138498	0.123763251	0.121350207	0.121744307	0.118174414	0.113835203	0.126505123	0.120879339	0.140727635	0.114610678
	12	0.117280298	0.123963996	0.116373867	0.147618235	0.123975491	0.122490225	0.120260683	0.119057856	0.140904159	0.114430459
	13	0.114765855	0.121666308	0.11494279	0.139563396	0.118524588	0.118209719	0.128668571	0.117709704	0.113165232	0.106407641
	14	0.118550451	0.138881931	0.112866373	0.120198694	0.126549459	0.116145207	0.126181632	0.127837265	0.112223086	0.118409642
	15	0.14207825	0.121214735	0.113107759	0.117861596	0.127060559	0.113279768	0.114433333	0.125197613	0.112662344	0.118591503
	16	0.123133922	0.119279537	0.119681438	0.124491927	0.121566552	0.121330912	0.121469668	0.114533089	0.123443045	0.118653492
	17	0.121570246	0.121105536	0.126955465	0.127201367	0.130609926	0.129050355	0.140107748	0.131328749	0.142276121	0.134777539
	18	0.126854066	0.115107819	0.117602968	0.115717033	0.116654253	0.117951501	0.111860596	0.115675981	0.116213764	0.127115158
	19	0.117391139	0.118166203	0.123947575	0.115827053	0.112801511	0.114143505	0.122254996	0.119402694	0.120625226	0.128459205
	20	0.112114708	0.118529104	0.115330732	0.120027918	0.120204442	0.121711055	0.112224318	0.126846266	0.11733654	0.118580419
	21	0.122509519	0.134526299	0.122476677	0.119791457	0.130815187	0.11740017	0.117968743	0.118235992	0.120471281	0.119172802
	22	0.119277485	0.125963235	0.118325486	0.126771962	0.137190583	0.119394073	0.119853446	0.127095042	0.122894177	0.124543652
	23	0.114362313	0.117882123	0.117590652	0.114779402	0.11621869	0.111419696	0.124857701	0.113460397	0.125692291	0.129497824
	24	0.108709434	0.120800108	0.119937603	0.119802952	0.128990419	0.117235141	0.120158053	0.12047046	0.125715691	0.119070171
	25	0.130760998	0.115466204	0.115221534	0.121048474	0.126810962	0.11903774	0.121685192	0.12202182	0.11787186	0.115377532

**SY eggs**	1	0.121670003	0.121718855	0.115248218	0.116349236	0.118796764	0.113473944	0.11945483	0.11821095	0.122014841	0.116302026
	2	0.113490365	0.112228833	0.118749554	0.116758115	0.107998	0.11766003	0.115462099	0.115858252	0.11184869	0.116900566
	3	0.113695215	0.124394633	0.121747181	0.133478239	0.128730149	0.120817761	0.129978544	0.131170288	0.12527438	0.126331473
	4	0.126712026	0.134716371	0.140138537	0.133333735	0.127861896	0.129415309	0.131552483	0.122537024	0.126278926	0.141042094
	5	0.120648215	0.122630623	0.118556199	0.116442014	0.105626419	0.11432085	0.11232859	0.114780634	0.116807788	0.109499687
	6	0.11520265	0.10306025	0.114215346	0.117509369	0.111341697	0.123980417	0.123386393	0.11171445	0.107883054	0.114672667
	7	0.1258331	0.122794831	0.121039442	0.119334958	0.115060609	0.116820104	0.107413418	0.116147259	0.12453421	0.126267842
	8	0.114231356	0.115487552	0.11850981	0.111598272	0.119545965	0.115311027	0.124136415	0.127711645	0.117032754	0.112813826
	9	0.111634398	0.11647034	0.117082016	0.115976072	0.1166756	0.111406149	0.113920592	0.115191566	0.117155089	0.109859715
	10	0.120441723	0.127443985	0.113442334	0.116749905	0.117524969	0.113537575	0.114791718	0.135268933	0.116878398	0.113666889
	11	0.138785048	0.107765235	0.110295688	0.114317976	0.111296129	0.11421042	0.116917808	0.111244814	0.124341265	0.109346152
	12	0.125303117	0.118362432	0.115760959	0.112366358	0.120003697	0.123308394	0.11315579	0.115277775	0.122496382	0.114828665
	13	0.111975542	0.136534159	0.124658188	0.107851444	0.110626979	0.112945604	0.117398118	0.113992022	0.11571498	0.114599594
	14	0.111264108	0.113819603	0.10768272	0.112613081	0.116616485	0.117727767	0.111209919	0.119662964	0.113767878	0.103938765
	15	0.113185758	0.107108811	0.109724653	0.140032212	0.142540086	0.108401953	0.110048965	0.11046277	0.11214755	0.114944432
	16	0.127720266	0.114361081	0.116642759	0.118035247	0.134890432	0.12050248	0.134484837	0.110513265	0.12180958	0.134744697
	17	0.111933258	0.109784179	0.112640586	0.111542852	0.113708763	0.11432085	0.110977975	0.106647796	0.115265049	0.125805184
	18	0.110452918	0.112716533	0.119944582	0.114267482	0.113867224	0.118617366	0.114055653	0.118104215	0.119010646	0.118334107
	19	0.119809931	0.120034896	0.123827292	0.116142333	0.110889713	0.113377061	0.114980147	0.118008563	0.132539787	0.117227341
	20	0.112731722	0.115509309	0.121985283	0.117184647	0.1119271	0.1193132	0.110654894	0.11903733	0.114571679	0.13899975
	21	0.10783256	0.137281719	0.11469114	0.117410023	0.116153007	0.113633637	0.116036419	0.113878718	0.116795473	0.134504952
	22	0.112746911	0.112885257	0.139253453	0.11342345	0.118411285	0.111615104	0.107944222	0.132718253	0.132290489	0.1075649
	23	0.119396536	0.112849952	0.123843303	0.113241589	0.118095594	0.116056534	0.118637482	0.11469894	0.121733223	0.123876145
	24	0.113742015	0.130393992	0.112910709	0.109851504	0.114772834	0.115057325	0.114268714	0.111021079	0.112088845	0.112346653
	25	0.125876615	0.122803452	0.114446059	0.111606072	0.120553795	0.114506816	0.119390788	0.116642759	0.112363895	0.135972156
**average**		0.12017214									
**std**		0.007983459									

The FLD-based algorithm											
		The wall clock time (s)									
		1	2	3	4	5	6	7	8	9	10
DY eggs	1	0.218055219	0.206228288	0.199755487	0.195833289	0.204282228	0.206178837	0.210061547	0.214901594	0.204068946	0.205599181
	2	0.205269532	0.210211987	0.203349191	0.201861384	0.200042364	0.202956244	0.206231162	0.198432299	0.205790073	0.20036216
	3	0.197380133	0.207204319	0.218317953	0.201025562	0.204920966	0.200983689	0.204390351	0.207440779	0.207555315	0.19422774
	4	0.196041833	0.201569503	0.208701901	0.221209665	0.21083639	0.219790903	0.204389452	0.211970472	0.201650376	0.209169074
	5	0.202320757	0.206357413	0.199361309	0.207293813	0.219914059	0.221215412	0.210153693	0.205257216	0.204336417	0.207438727
	6	0.198482793	0.201038289	0.207478547	0.204148809	0.212082544	0.208618154	0.198705706	0.204504509	0.20535451	0.198565719
	7	0.220657103	0.203457901	0.206586562	0.21641026	0.20995358	0.2091818	0.203042532	0.209980264	0.217327365	0.21207967
	8	0.212964754	0.203754487	0.206655641	0.2026703	0.204198892	0.211765211	0.213105974	0.203784676	0.203183673	0.207910194
	9	0.205851429	0.191959609	0.193910407	0.192902987	0.199431508	0.210884011	0.205517897	0.20253587	0.202210327	0.201469003
	10	0.198057904	0.194290549	0.198291901	0.201138045	0.204669539	0.2161348	0.209351945	0.19262917	0.202065002	0.201991519
	11	0.205242027	0.205262553	0.200988615	0.195174812	0.198184344	0.204867632	0.202183421	0.191622571	0.201910014	0.211159692
	12	0.20748881	0.200740328	0.203469806	0.192357404	0.198722948	0.196437165	0.196130917	0.197526279	0.190026464	0.194711334
	13	0.208407146	0.207100868	0.202940234	0.205832546	0.205689085	0.199851471	0.209419903	0.200319876	0.210671172	0.201670081
	14	0.204086787	0.207010963	0.198713917	0.197312807	0.206545921	0.203011254	0.209350114	0.197650256	0.208282126	0.219127911
	15	0.211188429	0.20845723	0.218324931	0.202918066	0.201448367	0.203886486	0.204854905	0.207019584	0.204557466	0.20343458
	16	0.19933832	0.20081127	0.202883771	0.196793498	0.202499822	0.200159773	0.21694599	0.201716548	0.202255074	0.207357932
	17	0.211403542	0.20674084	0.208541387	0.202811109	0.203200283	0.209600943	0.213708619	0.201752674	0.204207292	0.210346449
	18	0.202231264	0.195101329	0.220281887	0.196735204	0.198208154	0.221100466	0.198930672	0.203054026	0.20711852	0.210783244
	19	0.211176934	0.196552111	0.215837172	0.203926717	0.216790403	0.211498372	0.20085807	0.204902526	0.200783355	0.201725091
	20	0.196433471	0.202442682	0.205161565	0.217001821	0.205150891	0.201032952	0.208241706	0.198117019	0.194029458	0.201535019
	21	0.209876813	0.206442802	0.212626895	0.200756671	0.199681926	0.210769586	0.209514212	0.202533407	0.196873139	0.209121232
	22	0.207551398	0.210179367	0.208984339	0.204415237	0.21663071	0.202369609	0.203364491	0.199232816	0.207352295	0.204441921
	23	0.201799395	0.202920529	0.207226077	0.205153354	0.219530633	0.209895065	0.202773562	0.202111391	0.196582079	0.200547716
	24	0.207267617	0.197177746	0.213456559	0.205332941	0.201918036	0.201839216	0.200599441	0.21103243	0.202897129	0.2108602
	25	0.210799665	0.204280997	0.203331461	0.219822103	0.215068266	0.197380543	0.210498531	0.198078019	0.208250327	0.207456978

**SY eggs**	1	0.204136904	0.219909544	0.200760776	0.211563645	0.199664684	0.207437273	0.192973186	0.206349203	0.203418491	0.198947914
	2	0.197777518	0.200186046	0.200893375	0.193706234	0.194687524	0.194593292	0.197541057	0.208572997	0.193453907	0.197646151
	3	0.198044767	0.21647307	0.193962543	0.216468964	0.200257887	0.196426492	0.208422225	0.203462006	0.208770868	0.208107954
	4	0.207773302	0.218867641	0.192047871	0.215118349	0.200033743	0.207484705	0.192367257	0.195053709	0.220737155	0.198472941
	5	0.210767234	0.194036437	0.187548968	0.197931053	0.202141959	0.192437046	0.190081064	0.214353548	0.202972033	0.187727134
	6	0.185204481	0.203380091	0.198375647	0.195481882	0.21327634	0.202569311	0.210770518	0.198317764	0.192013244	0.194388664
	7	0.200747639	0.211818168	0.191995325	0.193144041	0.196878065	0.19423554	0.213587515	0.197777928	0.196825929	0.214427852
	8	0.198478766	0.19815766	0.192671043	0.192524076	0.207073962	0.199822735	0.203315861	0.204904168	0.193347549	0.189934508
	9	0.204453604	0.194381685	0.196340693	0.195437546	0.211321027	0.208930561	0.214154035	0.200090805	0.201708448	0.203494737
	10	0.207124678	0.212519749	0.213803039	0.196321809	0.20135581	0.197979494	0.210190451	0.197719224	0.220651766	0.195409631
	11	0.19518097	0.21694558	0.204455246	0.21063874	0.200599031	0.207258918	0.204766011	0.204311786	0.20288974	0.220879195
	12	0.19612599	0.211135882	0.195174402	0.213946721	0.190877886	0.194077078	0.19302286	0.194105404	0.199601464	0.194550409
	13	0.200586305	0.201870005	0.196490123	0.20291456	0.19841711	0.211840747	0.199346941	0.191788832	0.202956655	0.199949175
	14	0.198484025	0.205712484	0.197797223	0.197935979	0.196230263	0.207009321	0.192040071	0.218501866	0.191370101	0.194268381
	15	0.211990587	0.218889809	0.211494677	0.196433881	0.220105773	0.187681566	0.207403011	0.209079769	0.19520478	0.199516075
	16	0.208480819	0.20905536	0.196119011	0.203549448	0.199184785	0.200601083	0.202521691	0.190682067	0.198021778	0.193776987
	17	0.196709752	0.211722517	0.200624483	0.215261211	0.217684518	0.216036685	0.210184293	0.19727545	0.195016762	0.192890672
	18	0.199703684	0.218168523	0.199161385	0.219531043	0.215849488	0.204465731	0.19493671	0.195355852	0.204412363	0.201720575
	19	0.197217156	0.197032421	0.189948465	0.208078729	0.190812613	0.203499774	0.193157511	0.199030839	0.198722538	0.208983518
	20	0.193848828	0.193355792	0.193352508	0.198730748	0.205913008	0.196699078	0.190843402	0.200611757	0.198862115	0.202430777
	21	0.200101479	0.206599211	0.199487749	0.206842839	0.193852523	0.184325144	0.194607882	0.21428417	0.195494608	0.191181671
	22	0.197617004	0.196539385	0.211073072	0.220121373	0.213247193	0.205744095	0.194066405	0.211171375	0.205457961	0.190463259
	23	0.211212949	0.200440159	0.199656474	0.20518907	0.212935607	0.19888962	0.199304246	0.214248865	0.200025532	0.203841517
	24	0.196169506	0.210942526	0.204049873	0.205534507	0.196326325	0.196436755	0.200140068	0.21433138	0.213888838	0.198618676
	25	0.190846275	0.204478868	0.208025772	0.205285731	0.188404084	0.212747589	0.212180659	0.193030249	0.192209206	0.190276882
**average**		0.203467193									
**std**		0.007527247									
